# Measuring Health Equity in Emergency Care Using Routinely Collected Data: A Systematic Review

**DOI:** 10.1089/heq.2021.0035

**Published:** 2021-12-01

**Authors:** Kevin Morisod, Xhyljeta Luta, Joachim Marti, Jacques Spycher, Mary Malebranche, Patrick Bodenmann

**Affiliations:** ^1^ Department of Vulnerabilities and Social Medicine, Centre for Primary Care and Public Health (Unisanté), Lausanne, Switzerland.; ^2^ Faculty of Biology and Medicine, University of Lausanne, Lausanne, Switzerland.; ^3^ Department of Epidemiology and Health Systems, Centre for Primary Care and Public Health (Unisanté), Lausanne, Switzerland.; ^4^ Department of Medicine, University of Calgary, Calgary, Canada.

**Keywords:** health equity, emergency care, determinants of health

## Abstract

**Introduction:** Achieving equity in health care remains a challenge for health care systems worldwide and marked inequities in access and quality of care persist. Identifying health care equity indicators is an important first step in integrating the concept of equity into assessments of health care system performance, particularly in emergency care.

**Methods:** We conducted a systematic review of administrative data-derived health care equity indicators and their association with socioeconomic determinants of health (SEDH) in emergency care settings. Following PRISMA-Equity reporting guidelines, Ovid MEDLINE, EMBASE, PubMed, and Web of Science were searched for relevant studies. The outcomes of interest were indicators of health care equity and the associated SEDH they examine.

**Results:** Among 29 studies identified, 14 equity indicators were identified and grouped into four categories that reflect the patient emergency care pathway. Total emergency department (ED) visits and ambulatory care-sensitive condition-related ED visits were the two most frequently used equity indicators. The studies analyzed equity based on seven SEDH: social deprivation, income, education level, social class, insurance coverage, health literacy, and financial and nonfinancial barriers. Despite some conflicting results, all identified SEDH are associated with inequalities in access to and use of emergency care.

**Conclusion:** The use of administrative data-derived indicators in combination with identified SEDH could improve the measurement of health care equity in emergency care settings across health care systems worldwide. Using a combination of indicators is likely to lead to a more comprehensive, well-rounded measurement of health care equity than using any one indicator in isolation. Although studies analyzed focused on emergency care settings, it seems possible to extrapolate these indicators to measure equity in other areas of the health care system. Further studies elucidating root causes of health inequities in and outside the health care system are needed.

## Introduction

Equity is defined by the World Health Organization as “the absence of avoidable, unfair, or remediable differences among groups of people, whether those groups are defined socially, economically, demographically, or geographically or by other means of stratification.”^1^ Applied to health care, equity means guaranteeing the “distribution of care in such a way as to get as close as feasible to an equal distribution of health.”^2^

These definitions imply two essential components of equity: horizontal equity (same care for the same health need) and vertical equity (different care for different needs).^3^ To be able to analyze equity within the health care system, most researchers assume that vertical equity is on average satisfied and focus their analysis on horizontal equity, that is, inequalities in the use of the health care system for the same health needs.^4^

However, achieving equity in health care remains a challenge for health care systems worldwide.^5–7^ Several recent studies raise the importance of addressing the concept of equity when making decisions about health care policies and practices.^8–10^ However, the performance assessment of health care systems has traditionally been limited to quality and efficiency indicators and health care decision makers remain poorly informed about equity,^8^ particularly in some specific settings, such as emergency care.^10^ Measuring and monitoring equity is therefore an emerging area of interest in assessing emergency care performance.^10–13^

Emergency care is a unique health care setting as it is situated at the interface of outpatient (ambulatory) care and inpatient (hospital based) care. Identifying indicators of health care equity in this setting makes it possible to assess both access to outpatient care, while also highlighting differences in quality of care within hospital-based care.^14,15^

To ensure accessibility of quality data on relevant variables for measuring health care equity, several approaches and data could be used, from primary qualitative or quantitative data to the use of routinely collected administrative data. For this study, we have decided to focus on studies based upon routinely collected administrative data as it has two fundamental advantages in the analysis of health care equity: the achievement of near complete coverage of the target population and the possibility of disaggregation in subpopulations. Moreover, using administrative data minimizes cost and burden of response.^16^

Finally, we have focused our analysis on studies measuring equity through socioeconomic determinants of health (SEDH), that is, the level of education, financial resources, and social and material living conditions.^17,18^

The aim of this systematic review is to identify how health care equity is measured through the combination of administrative data-derived emergency care equity indicators and SEDH with the goal of creating a set of valuable and replicable indicators that can be used in the identification and analysis of health care equity in emergency care settings.

## Methods

The protocol of this systematic review was published in PROSPERO at the outset of the study (Supplementary File S1). The reporting of this systematic review was based on the PRISMA-equity guidelines^19^ (Supplementary File S2).

### Inclusion/exclusion criteria

We included studies reporting on health care equity indicators, which were analyzed as such, focusing on studies that used administrative data and were conducted in emergency care settings. This included several study designs, such as retrospective cohort studies, cross-sectional studies, and ecological (small-area level) studies. As this systematic review's objective is to focus on health care equity in the context of emergency care and not to identify inequalities in emergency care provision between countries, a focus was placed on studies conducted in high-income countries.

It is indeed tricky, in countries where health care resources are often lacking or insufficient, to determine whether variations in the use of care among specific populations are linked to inequities in access to care or whether they are the result of an overall lack of resources in the health care system. We included studies on adults (age 18 and over). If a study included both children and adults, we limited data extraction to data pertaining only to adults. We included studies regardless of whether a disease-specific focus was taken (e.g., cancer, chronic diseases, or mental health). Searches were limited to English, German, French, and Italian (due to the authors' language skills), published between January 2010 and January 2019.

We chose to focus on studies published after 2010 because of the significant evolution of health care equity-related literature that followed the WHO Report “Closing the gap in a generation: Health equity through action on the social determinants of health.”^20^

We limited our analysis to studies looking at inequities and their associated SEDH as defined above, excluding studies looking at determinants of health such as race/ethnicity, gender, or place of residence, to ensure consistency and comparability between studies and countries.^4,18^

We excluded studies that did not focus on equity, as well as opinion articles, editorials, conference abstracts, and study protocols.

### Search strategy

The search strategy was conducted with a medical librarian's assistance using four databases: Ovid MEDLINE, EMBASE, PubMed, and Web of Science. We used keywords in the field of equity, socioeconomic factors, and emergency care. We combined the Medical Subject Headings terms “Health Services Accessibility,” “Health Equity,” or “Health care Disparities” with a combination of terms defining administrative data and with text words “emergency department” or “emergencies.” Initial searches were conducted in November 2018 to assess the scope of the literature. The last search was conducted in January 2019. The full search strategy can be found in Supplementary File S3.

Following the initial search, we screened reference lists of all included studies and performed Google and Google Scholar searches using key search terms to identify any further relevant studies that were not initially captured or had not yet been published.

### Study selection

Two reviewers (K.M. and X.L.) conducted screening of articles independently and in duplicate. This was done in two stages. First by screening all titles and abstracts and second, by reviewing the full text of all relevant articles to determine their eligibility in the final analysis. Two other reviewers (J.M. and P.B.) provided arbitration in the event of a disagreement at both stages of screening. Reasons for exclusion of articles at the full-text screening stage were documented.

### Data extraction

Two authors (K.M. and X.L.) extracted data independently and in duplicate from included studies using Rayyan^®^* and any discrepancy was resolved by consulting the two other reviewers (J.M. and P.B.). Data on the key characteristics of the studies were extracted in a predefined data extraction form, into an Excel^®^ spreadsheet.^†^

### Quality and bias assessment

Risk of bias was assessed using the validated checklist published by the United States National Heart, Lung and Blood Institute (NIH) for observational cohort and cross-sectional studies.^21^ This tool is composed of 14 questions. It has been recently recommended in a review for the assessment of both observational cohort and cross-sectional studies.^22^

## Results

**FIG. 1.**
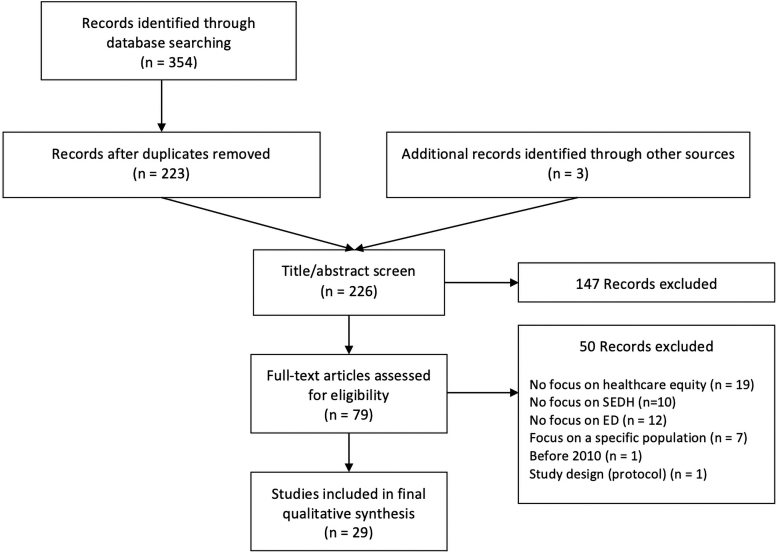
Flow diagram of literature research.

The initial search yielded 354 articles, of which 29 were included in the final analysis (Fig. 1). Of these, 17 (59%) were conducted in the United States, 5 (17%) in the United Kingdom, 3 (10%) in Canada, 2 (7%) in Australia, 1 (3%) in Sweden, and 1 (3%) in Switzerland. Twenty-eight (97%) were written in English and one (3%) in French.

### Risk of bias assessment

The NIH quality and risk of bias assessment tool used made it possible to evaluate the internal validity of selected studies in this review. Of the 29 studies, 28 are considered fair, and 1 study is considered poor, mainly due to the lack of statistical analysis of confounding factors. The detailed assessment is available in Supplementary Materials (Supplementary Table S1).

Moreover, the bias assessment revealed two significant risks of bias across studies. First, there is a risk for confounding related to the use of retrospectively collected administrative data used across all included studies as adjustment can only be performed with available collected variables. For example, the almost systematic absence of precise clinical diagnoses in administrative data undermines the ability to estimate the health outcomes of selected populations accurately.

Second, comparisons between studies are biased because, for the same variable, data are not collected in a standardized manner. This information bias is particularly relevant for the assessment of social deprivation, often analyzed using indices that include many variables that differ between studies.

The significant heterogeneity associated with a large number of outcomes and exposures prevented the authors from performing a meta-analysis.

### Equity indicators

The analysis of the 29 articles highlighted 14 different indicators used to assess health care equity. We categorized them into four groups according to the part of the patient care pathway they analyzed:

A. Equity indicators of poor access to outpatient care (indicators “before emergency care”) (Group 1)
B. Equity indicators of quality of emergency care (indicators “during emergency care”) (Group 2)
C. Equity indicators of clinical outcomes (indicators “following emergency care”) (Group 3)
D. Global Equity indicators (Group 4)

#### Equity indicators of poor access to outpatient care (Group 1)

1. ED visits/emergency admissions^‡^ rate

With 26% (*n*=7) of articles using this indicator, it was the most commonly reported indicator identified in this systematic review.^23–29^ It was used to highlight disparities of access to outpatient care.

2. Ambulatory care sensitive conditions (ACSCs)^§^ ED visits/ACSC emergency admission rate

Also called Preventable ED visits/Preventable emergency admissions, this indicator, used in seven articles, is used as often as the previous indicator “ED visits/emergency admission rate.”^10,24,26,30–33^

3. Frequent ED visits

One study used this indicator considering frequent ED visits when 4 or more ED visits occurred by an individual per year.^34^

4. ED-associated initial diagnosis rate

This indicator compared the rate of initial diagnosis of cancer in the ED between different SEDH.^35^

#### Equity indicators of quality of emergency care (Group 2)

5. Emergency-specific procedure rate

This indicator comprised a combination of different procedures performed during emergency care, highlighting disparities in the quality or access to care for specific emergency conditions such as a brain scan for the diagnosis of acute stroke,^36^ reperfusion therapy in acute stroke,^37^ and cardiac catheterization after myocardial infarction or cardiac arrest.^38,39^

6. Delay to diagnosis or treatment rate

Two studies focused on disparities in time to access to a diagnostic,^36^ or therapeutic procedure.^40^

7. Missed diagnoses in ED rate

This indicator, used in one study, highlighted disparities of missed diagnoses of acute myocardial infarction according to insurance status or median household income.^41^

#### Equity indicators of outcome after emergency care (Group 3)

8. Major adverse event rate

This indicator was used in two studies that analyzed emergency general surgery.^42,43^ It represented the rate of specific complications following an emergency general surgery.^**^

9. In-hospital mortality and (10) failure to rescue rate

In-hospital mortality was used to reflect the quality of care during emergency care or surgery as reported in three articles identified in our review.^39,42,43^ One distinguishes in-hospital mortality from failure to rescue.^42^

11. Neurological recovery rate

This specific indicator was used in one study analyzing the neurological recovery over time of patients who presented to the ED with a cardiac arrest.^39^

12. Length of stay/Bed days (after emergency admission)

Although these are traditional indicators of hospital care quality, they are used in one study that analyzed inequities following emergency admission according to social deprivation.^44^

#### Global equity indicators

13. 30-/90-/365-day mortality rate

One study analyzed 30-/90-/365-day mortality following emergency admission for hip fracture, reflecting quality of ED- and hospital-based care, as well as access to and quality of ambulatory follow-up care post-discharge.^45^

14. ED readmission rate/Emergency rehospitalization rate

This indicator was used in three articles. Two of them analyzed ED readmissions within 30 days post-discharge.^46,47^ One used this indicator to analyze the rate of hospital admissions through the ED in the year following a diagnosis of cancer.^48^

The different emergency care equity indicators are summarized in Table 1.

**Table 1.** Emergency Care Equity Indicators

Group 1	Group 2	Group 3
Access to high-quality outpatient care (i.e., before ED care)	Quality of emergency care (i.e., during ED care)	Outcome following emergency care (i.e., after ED care)
ED visits/emergency admission rate	Specific procedure rate (including management of STEMI, ischemic stroke, and out-of-hospital cardiac arrest)	MAE rate (specifically following emergency surgery)
Preventable ED visits/preventable emergency admission rate (ACSCs)^a^	ED missed diagnosis rate	In-hospital mortality rate/failure to rescue rate (after emergency admission)
ED-associated initial diagnosis rate (of cancer, in “emergency presenters”)	Delay to diagnosis or treatment rate (for emergency conditions)	Recovery rate (after out-of-hospital cardiac arrest)
Frequent ED visit rate (four or more a year)		LOS/bad days (after emergency admission)
ED readmission rate/emergency rehospitalization rate (within 30 days of discharge or during the year after diagnosis of cancer)		
30-/90-/365-day mortality rate (specifically following emergency hip fracture admission)		

^a^ ACSCs: conditions for which timely and appropriate outpatient care can prevent disease complications, more severe disease, or need for hospitalization.

ACSCs, ambulatory care sensitive conditions; ED, emergency department; LOS, length of stay; MAEs, major adverse events; STEMI, ST segment elevation myocardial infarction.

### Socioeconomic determinants of health

The articles included in this review analyzed health care equity based on seven SEDH:

Insurance status, social deprivation, income, education level, social class, health literacy, and financial and nonfinancial barriers (see Table 2 for details).

**Table 2.** Description of the Selected Articles

Authors (year of publication) Country	Study design	Aim	Population	Emergency care indicators	Socioeconomic determinants of health	Statistics	Key conclusion
Insurance status							
Berlin et al. (2016) Switzerland	Nation-wide cross-sectional study	To determine if patient characteristics affected the rate of revascularization in acute STEMI patients	Patients with acute STEMI (*n*=9696)	Specific procedures Revascularization rate	Insurance status (ref: public) a. Half Private b. Private	Relative proportion): a. 1.05 (95% CI 0.98–1.13) b. 1.06 (95% CI 0.96–1.17)	No association was found between insurance status and revascularization rate.
Bradshaw et al. (2015) Australia	Population-based cohort study	To determine whether quality indicator-based outcomes of PPM implantation were comparable for publicly and privately insured patients	Adults implanted with a PPM between 1995 and 2009. (*n*=9748)	Specific procedures 1. Emergency implant of PPM (%) Delay to treatment 2. Time to PPM implementation for emergency cases 3. LOS for emergency cases (≥2 days)	Insurance status (public vs. private)	1. 60% vs. 33%, (*p < 0.001*), nonadjusted 2. Adjusted odds ratio 0.89 (95% CI 0.78–1.03) (*p*=*0.11*) 3. Adjusted odds ratio 0.91 (95% CI 0.79–1.04) (*p*=*0.17*)	Publicly insured patients are more likely to have emergency implant of a PPM. There were no differences identified in outcomes between publicly and privately insured patients.
Casey and Mumma (2018) United States	Retrospective cohort study	To evaluate the association of patient insurance status with hospital treatments and outcomes following out-of-hospital cardiac arrest	Adult patients with a “present on admission” diagnosis of cardiac arrest (patient admitted from the ED to an acute care hospital) (*n*=38,163)	1. Good neurologic recovery 2. In-hospital mortality (survival to hospital discharge) Specific procedures 3. Cardiac catheterization 4. DNR within 24 h 5. Treatment at 24/7 PCI^a^ center (STEMI center)	Insurance status (ref: private insurance) a. Medicare insurance b. Government insurance	Odds ratios with 95% CI 1.a. 0.85 (0.79–0.91) 1.b. 0.94 (0.88–1.01) 2.a. 0.78 (0.73–0.83) 2.b. 0.65 (0.61–0.69) 3.a. 1.25 (1.15–1.36) 3.b. 1.24 (1.13–1.36) 4.a. 0.44 (0.40–0.48) 4.b. 0.56 (0.51–0.61) 5.a. 0.87 (0.82–0.94) 5.b. 0.91 (0.85–0.98)	Insurance status is independently associated with the likelihood of good neurological recovery, survival to hospital discharge, treatment at a 24/7 PCI center, receiving a DNR order within 24 h of admission and undergoing cardiac catheterization in patients experiencing out-of-hospital cardiac arrest.
Davis et al. (2010) United States	Retrospective cohort study	To examine the frequency of return visits for treating dental health problems in hospital emergency rooms for patients without access to private dental services	Individuals without access to private dental services (*n*=7846)	ED visits (dental-related)	Insurance status (private, commercial), public, Medicare or self-pay)	—	Patients without insurance are much more likely to resort to the ED for dental problems than those with private coverage
DeLeire et al. (2010) United States	Case-crossover study	To examine if expanding Medicaid to low-income childless adults impacts ED use	Low-income childless adults (*n*=9619)	1. Total ED visits 2. ACSCs ED visits 3. Specific ED visits (for mental health/ drug/alcohol) 4. Unclassified ED visits	Insurance status (comparison before and after introduction of public insurance)	Predicted increase after introduction of insurance (%): 1. 46%, *p*<0.01 2. 38.7%, *p*<0.01 3. 343.9%, *p*<0.01 4. 89.8%, *p*<0.01	Public insurance coverage expansions to childless adults have the potential to improve health and reduce costs by increasing access to outpatient care, ED visits, and reducing hospitalizations.
Kerr et al. (2014) United States	Retrospective cohort study	To determine factors associated with ED utilization for HIV-infected patients	HIV-infected South Carolina residents (*n*=4947)	Total ED visits	Insurance status (ref: private) a. Self-pay b. Medicare c. Medicaid d. Indigent/charity e. HMO	IRR (95% CI) a. 0.65 (0.61–0.70) b. 0.88 (0.82–0.95) c. 1.26 (1.18–1.36) d. 1.65 (1.47–1.86) e. 1.08 (0.95–1.23)	Insurance type is associated with ED utilization. There is a need to evaluate HIV primary care systems to increase access and develop interventions to reduce preventable ED visits.
Lines et al. (2019) United States	Retrospective cohort study	To compare PCS conditions ED use for public vs. private insurees	People younger than 65 years in the Massachusetts all-payer claims data (*n*=2,269,455)	Preventable ED visits	Insurance status (ref: private) Any public insurance	Rate ratio (95% CI) 2.53 (2.49–2.56)	Public insurance is associated with more PCS ED use. Statewide labor shortages and low reimbursement rates from public insurance may provide inadequate access to care that may otherwise help reduce PCS ED use.
Livingood et al. (2016) United States	Retrospective, population-based study	To clarify some of the factors associated with the use of ED for initial cancer diagnoses	Patients with a primary or any secondary diagnosis of cancer (*n*=989)	ED-associated initial diagnoses (of cancer)	Insurance status (ref: private) a. Medicaid b. Medicare c. Uninsured	Relative risk (95% CI) a. 3.10 (1.87–5.39) b. 4.35 (2.63–7.54) c. 2.67 (1.60–4.65)	There is a significant relationship between health insurance and ED-associated initial cancer diagnosis.
Mazurenko et al. (2010) United States	Retrospective pre-/post-cohort study	To examine the impact of Nevada's Medicaid expansion on changes in rates of hospital ED admissions for ACSCs	Patient hospitalized a. Pre-expansion of Medicaid (*n*=107,940) b. Post-expansion of Medicaid (*n*=106,016)	ACSC emergency hospital admission	Insurance status (Medicaid or uninsured)	Odds ratio (95% CI) a. 0.50 (0.16–0.83) b. 0.62 (0.29–0.94)	Uninsured patients are more likely to be admitted through the ED for ACSCs, regardless of Medicaid expansion.
Metcalfe et al. (2018) United States	Retrospective cohort study	To identify socioeconomic disparities in EGS and whether they are more likely to be associated with MAEs or an FTR appropriately to such events	Patients presenting EGS with acute surgical conditions (*n*=1,345,199)	1. MAEs^b^ 2. In-hospital mortality 3. FTR^c^	Insurance status (ref: private) a. Public b. Uninsured	Odds ratio (95% CI) 1.a. 1.18 (1.16–1.20) 1.b. 1.16 (1.13–1.19) 2.a. 0.96 (0.92–1.01) 2.b. 1.28 (1.16–1.41) 3.a. 1.01 (0.95–1.07) 3.b. 1.20 (1.06–1.36)	Lack of insurance is an independent risk factor for in-hospital mortality, due to both excess MAEs and FTR.
Social deprivation							
Cookson et al. (2018) United Kingdom	Whole-population study at the small-area level	To present a new and improved analytical approach to integrating health equity into mainstream health care quality assurance	CCG-LSOA^d^ (CCGs, *n*=209) (LSOAs, *n*=32,844)	Potentially avoidable emergency admissions	Social deprivation IMD (between the most and least deprived neighborhoods)	Absolute gradient index (95% CI) 927/100,000 (912–942) (the mean rate of potentially avoidable emergency admissions: 792/100,000 people)	Administrative data on inequality in health care quality within similar populations served by different health care organizations can provide useful information for health care quality assurance.
Fairley et al. (2011) Scotland (United Kingdom)	Longitudinal retrospective analysis between 1980 and 2000	To examine whether individual social class, area deprivation, or both are related to emergency Caesarean sections in Scotland and investigate changes over time	Women with live singleton birth A. 1980–1981 (*n*=133,555) B. 1990–1991 (*n*=128,933) C. 1999–2000 (*n*=102,285)	Emergency admission (for Caesarean section)	a. Social class (divided in six classes) Social deprivation b. Index of area deprivation (Carstairs score)^e^	Odds ratio (95% CI) A.a. 1.14 (1.04–1.25) A.b. 1.18 (1.05–1.32) B.a. 1.13 (1.04–1.23) B.b. 1.13 (1.02–1.26) C.a. 1.02 (0.93–1.12) C.b. 1.02 (0.93–1.13)	In 1980–1981 and 1990–1991, both individual social class and area deprivation were associated with emergency C-sections. In 1999–2000, there was no significant association.
Lazzarino et al. (2011) United Kingdom	Retrospective cohort study	To identify any stroke patient groups being excluded from appropriate use of brain imaging based on levels of social deprivation	Patients with a principal emergency admission diagnosis of stroke (*n*=209,174)	Specific procedure 1. Brain scan, at any time 2. Brain scan on the same day of admission	Social deprivation Index of area deprivation (Carstairs score, in five quartiles)^f^ (ref: least deprived) a. Second b. Third c. Fourth d. Most deprived	Odds ratio (95% CI) 1.a. 0.98 (0.92–1.05) 1.b. 0.99 (0.93–1.06) 1.c. 0.99 (0.93–1.06) 1.d. 0.97 (0.91–1.04) 2.a. 0.95 (0.90–1.00) 2.b. 0.93 (0.88–0.98) 2.c. 0.91 (0.86–0.96) 2.d. 0.94 (0.89–0.99)	More-deprived patients have less chance of being scanned in a timely manner.
Levin and et Crighton (2017) United Kingdom	Ecological small-area study (during the course of RCOP^g^ program)	To examine mean LOS and rates of emergency bed days during the RCOP in Glasgow City	Data zones of household residents of Glasgow City 65 years of age and older	1. Bed days (after emergency admission) 2. LOS (after emergency admission)	Social deprivation Index of area deprivation SIMD^h^ (ref: SIMD 1, most deprived) a. SIMD 2 b. SIMD 3 c. SIMD 4 d. SIMD 5 (least deprived)	Relative risks (95% CI) 1.a. 0.89 (0.87–0.91) 1.b. 0.79 (0.76–0.81) 1.c. 0.67 (0.65–0.69) 1.d. 0.60 (0.58–0.62) 2.a 1.03 (1.01–1.04) 2.b. 1.01 (0.99–1.02) 2.c. 0.99 (0.97–1.004) 2.d. 0.96 (0.94–0.98)	The rate of emergency bed days rose with increasing deprivation, while no significant inequalities were observed for LOS.
Lines et al. (2017) United States	Retrospective cohort study	To explore associations between ED use and neighborhood poverty	Patients with commercial insurance (*n*=64,623)	1. Total ED visits 2. Preventable ED visits	Social deprivation (percent living in poverty in CT/10^i^)	*z*-score (*p*-value) 1. 5.84 (<0.01) 2. 6.2 (<0.01)	People in lower-income neighborhoods remain more likely to go to the ED, have more ED visits, and have more PCS ED visits than people in higher-income neighborhoods.
Shulman et al. (2018) Canada	Population-based cohort Study	To determine if the combination of socioeconomic status and mental health visits in adolescence is associated with diabetes-related ED visits in early adulthood	Patient with a diagnosis of diabetes before their 15th birthday (*n*=8491)	ED visits (diabetes-related)	Social deprivation Index of Marginalization area (based on the ON-MARG^j^, divided in five quintiles) (ref: least deprived)	Rate ratio (95% CI) 3.15 (1.79–5.54)	Socioeconomic status combined with mental health visits is associated with an increase in risk of diabetes-related ED visits in early adulthood for people with childhood-onset diabetes.
Thorne et al. (2016) United Kingdom	Record linkage study	To identify whether social deprivation has any effect on mortality risk after emergency admission with hip fracture	Patients emergency admitted with hip fracture in England (*n*=455,862) and Wales (*n*=29,733)	1. 30-day mortality (following hip fracture) 2. 90-day mortality (following hip fracture) 3. 365-day mortality (following hip fracture)	a. Social deprivation (2007 IMD, cf footnote l) for England, divided in five quintiles) (ref: least deprived) Most deprived b. Social deprivation (2008 Welsh Index of Multiple Deprivation)^k^ for Wales, divided in five quintiles) (ref: least deprived) Most deprived	Odds ratio (95% CI) 1.a. 1.187 (1.147–1.228) 1.b. 1.136 (0.991–1.302) 2.a. 1.185 (1.154–1.217) 2.b. 1.135 (1.022–1.261) 3.a. 1.154 (1.128–1.181) 3.b. 1.203 (1.100–1.317)	There is a positive association between social deprivation and increased mortality at 30 days post-admission for hip fracture in both England and Wales. This association is still evident at 90 and 365 days
Vanasse et al. (2012) Canada	Retrospective cohort study	To compare ED use in patients with mood disorder based on the dwelling sector level of material and social deprivation	Patients 18 years of age or older hospitalized with a diagnosis of mood disorder (*n*=177,850)	Total ED visits (during the year following the diagnosis of mood disorder)	Social deprivation Combination of material and social deprivation quintiles based on the INSPQ deprivation index^l^ (ref: least deprived) Most deprived a. Women b. Men	Relative risk a. 3.82 b. 3.25	There is a gradient between the level of disadvantage in the neighborhood of residence and the rate of ED visits.
Vanasse et al. (2014) Canada	Retrospective cohort study	To measure and compare ED use in relation to the level of material and social deprivation of the area of residence	Patients 30 years of age or older with diagnosis of hypertension without diagnosis of CVD (*n*=276,793)	Frequent ED visits (four or more visits per year)	Social deprivation (INSPQ deprivation index)^l^ (ref: least deprived) Most deprived	Relative risk 1.47	The risk of being frequent users is 47% higher for people living in the most materially and socially deprived areas than for people living in the least deprived areas.
Insurance status and social deprivation							
Whitney et al. (2017) United States	Retrospective cohort study	To examine individual predictors of rehospitalization among individuals with advanced cancer	Patients diagnosed with advanced breast, colorectal, non-small-cell lung, or pancreatic cancer (*n*=25,032)	Rehospitalizations (among individuals with advanced cancer in the year after diagnosis)^m^	a. Social deprivation (area-based SES quintile)^n^ (ref: highest) i. Upper-middle ii. Middle iii. Lower-middle iv. Lowest b. Insurance status (ref: private) i. Public ii. Uninsured	IRR (95% CI) a.i. 1.09 (1.02–1.18) a.ii. 1.13 (1.05–1.22) a.iii. 1.14 (1.05–1.24) a.iv. 1.29 (1.18–1.42) b.i. 1.37 (1.23–1.47) b.ii. 1.17 (1.02–1.35)	Rehospitalization rates are significantly associated with sociodemographic characteristics, such as insurance status and socioeconomic quintile.
Income							
Singhal et al. (2016) United States	Retrospective cohort study	To determine the factors associated with a subsequent dentist visit after a dental ED visit	Adults enrolled in Medicaid (*n*=2430)	Subsequent dentist visit after a dental ED visit	Reportable income (ref: no) a. Yes	Hazard ratio (95% CI) a. 1.05 (0.94–1.18)	No effect of reportable income on subsequent dentist visits was found after a dental ED visit among adults enrolled in Medicaid.
Insurance status and income							
Finnegan et al. (2017) United States	Observational, population-based study	To determine what factors are associated with an increased risk of ED visits following major joint replacement surgical procedures	Adult undergoing total hip or knee arthroplasty (*n*=152,783)	1. Total ED readmission (following intervention, within 30 days) 2. Specific ED readmission (following intervention, pain related, within 30 days)	a. Insurance status (ref: private) i. Medicare ii. Medicaid b. Median household income (ref: highest quartile) i. Second ii. Third iii. Fourth	Odds ratio (95% CI) 1.a.i. 1.38 (1.29–1.47) 1.a.ii 2.28 (2.04–2.55) 1.b.ii 0.98 (0.91–1.05) 1.b.iii 0.96 (0.89–1.03) 1.b.iv 0.97 (0.90–1.05) 2.a.i 1.62 (1.40–1.87) 2.a.ii 1.68 (1.36–2.09) 2.b.i 1.12 (0.96–1.31) 2.b.ii 1.00 (0.85–1.17) 2.b.iii 1.04 (0.87–1.23)	Medicaid patients had almost double the risk of an ED or pain-related ED visit following a surgical procedure. No association between median household income quartile and increased risk for an ED visit was found.
Ladha et al. (2011) United States	Retrospective cohort study	To determine whether re-presentation to ED after discharge from hospital is related to insurance status and socioeconomic factors such as neighborhood income level	Trauma patients (*n*=6675)	Total ED readmission (re-presentation to the ED within 30 days of discharge)	a. Insurance status (ref: private) i. Public ii. Uninsured b. Median household income (ref: >40,000 $) i. 20,000–40,000 $ ii. <20,000 $	Odds ratio (95% CI) a.i 1.64 (1.30–2.06) a.ii 1.60 (1.20–2.14) b.i 1.42 (1.14–1.77) b.ii 1.77 (1.37–2.29)	Re-presentation to ED is associated with being uninsured or underinsured and with lower neighborhood income level.
Moy et al. (2014) United States	Retrospective cross-sectional analysis	To identify factors associated with the frequency of missed AMI diagnosis in the ED	Patient evaluated for chest pain or cardiac conditions within 1 week of hospitalization (*n*=111,973)	ED missed diagnoses^o^ (of AMI)	a. Insurance status (ref: private) i. Medicare ii. Medicaid iii. Uninsured b. Median household income (ref: highest) i. Moderate ii. Low iii. Lowest	Odds ratio (*p*-value) a.i. 0.801 (*p*=0.0389) a.ii. 1.124 (*p*=0.3938) a.iii 0.871 (*p*=0.2798) b.i. 1.067 (*p*=0.6111) b.ii. 1.006 (*p*=0.9606) b.iii. 0.906 (*p*=0.4550)	The associations between missed diagnoses and expect payers (other than Medicare) and household income were not significant when controlling for other demographic and clinical conditions.
Shah et al. (2015) United States	Retrospective population-based cohort study	To determine the predictors of in-hospital complications and mortality among EGS patients	Patient 16 years of age and older with primary diagnosis and subdiagnosis of an EGS condition^p^ (*n*=32,910,446)	1.MAEs 2. In-hospital mortality	a. Insurance status (ref: private) i. Government ii. Uninsured b. Median household income quartile (ref: lowest) i. Second ii. Third iii. Fourth	Odds ratio (95% CI) 1.a.i. 1.15 (1.14–1.15) 1.a.ii 1.06 (1.04–1.08) 1.b.i. 1.01 (1.00–1.02) 1.b.ii. 1.03 (1.02–1.04) 1.b.iii 1.00 (1.00–1.02) 2.a.i. 1.08 (1.06–1.10) 2.a.ii 1.25 (1.20–1.30) 2.b.i. 0.98 (0.96–0.99) 2.b.ii. 0.92 (0.90–0.93) 2.b.iii. 0.86 (0.84–0.88)	Uninsured patients were at higher risk for death compared to government- or private insured patients. Patients in the highest income quartile had the least likelihood of mortality after an EGS condition.
Education level with/without insurance status							
Yap et al. (2018) Australia	Retrospective cohort study	To examine patients' characteristics associated with presenting to ED around the time of diagnosis	Patient newly diagnosed with non-small cell lung cancer (*n*=647)	“Emergency presenters” (presenting to an ED around the time of diagnosis)	a. Education level (ref: no school certificate) i. School certificate ii. Trade/Certificate/ Diploma/HSC iii. University degree b. Insurance status (ref: private) Not private	Odds ratio (95% CI) a.i. 0.97 (0.58–1.63) a.ii. 0.67 (0.41–1.11) a.iii 0.49 (0.24–0.99) b.1.28 (0.86–1.90)	The risk of being an “emergency presenters” seems to follow an educational-level gradient.
Stecksén et al. (2014) Sweden	Retrospective cohort study	To test whether patient education level is associated with receiving reperfusion treatment	Patients with ischemic stroke (*n*=85,885)	Specific procedure Reperfusion therapy	Education level (ref: primary) a. Secondary b. University	Odds ratio (95% CI) a. 1.08 (1.00–1.17) b. 1.14 (1.03–1.26)	Reperfusion therapy for stroke is associated with higher patient education level.
Health literacy							
Balakrishnan et al. (2017) United States	Observational cross-sectional study	To determine the association of health literacy with preventable ED visits	Adults and English-speaking patients. (excluded patients with impaired vision, hearing problems, being in police custody, or being too ill to participate) (*n*=1201)	1. Total potentially preventable ED visits 2. Potentially preventable ED visits resulting in hospital admission 3. Potentially preventable treat-and-release ED visits	Health literacy (assessed by the REALM^q^) Limited (REALM <61) vs. adequate (REALM ≥61) health literacy	Rate ratio (95% CI) 1. 1.93 (1.55–2.40) 2. RR 2.33 (95% CI 1.75–3.1) 3. RR 1.42 (95% CI 0.99–2.40)	Limited health literacy is a risk factor for potentially preventable ED visits.
Financial and nonfinancial barriers							
Shippee et al. (2014) United States	Cross-sectional study	To examine the distinct associations financial and nonfinancial barriers to care have with patterns of ED use among a publicly insured population	Publicly insured patients (*n*=1737)	ED visits (0, 1 or 2+ ED visits in 1 year)	a. Financial concerns^r^ b. Nonfinancial barriers^s^	Odds ratio (95% CI) a. 0.939 (0.849–1.038) b. 1.210 (1.048–1.398)	Nonfinancial barriers are associated with actual ED visits.

A *p*-value <0.05 is considered significant.

A *p*-value <0.001 is considered highly significant.

^a^ Percutaneous coronary center.

^b^ MAEs identified from ICD-9-CM codes (cerebrovascular accident, pneumonia, pulmonary embolus, acute respiratory distress syndrome, renal failure, urinary tract infection, myocardial infarction sepsis, septic shock, and cardiac arrest).

^c^ FTR: The odds of in-hospital mortality after an MAE.

^d^ CCG-LSOA: A block of CCG registered population residing within a neighborhood census unit called LSOA.

^e^ Carstairs score: An index of deprivation used in spatial epidemiology, based on four variables (male unemployment, lack of car ownership, overcrowding, and low social class).

^f^ Quintile of socioeconomic deprivation (Carstairs): a geographically based deprivation score based on four census indicators (low social class, lack of car ownership, overcrowding, and male unemployment).

^g^ RCOP: Program developed to address the projected increase in health service and social care use by older people in Scotland.

^h^ SIMD: The Scottish Government's official tool for identifying those places in Scotland suffering from multiple deprivation. By identifying concentrations of multiple deprivation, the SIMD can be used to target policies and resources at the places with greatest need.

^i^ CT/10: a coefficient that refers to the effect of a 10% increase in the percentage of the population in the CT who have household incomes below 200% of the federal poverty threshold. (The poverty coefficient indicates the effect of a 10% increase in the fraction of the population living in poverty).

^j^ ON-MARG: a validated census- and geography-based index that measures marginalization at the level of the census DA, including economic, ethno-racial, age-based, and social marginalization.

^l^ A composite score originates from the following domain indices: income, employment, health, education, access to services, community safety, and physical environment.

^l^ INSPQ deprivation index: an index based on six socioeconomic indicators calculated at the DA level. This index has two components, material and social. The material component is based on the proportion of people without a high school diploma, the employment-to-population ratio, and the average income. The social component is based on the proportion of people living alone, the proportion of separated, divorced, or widowed people, and the proportion of lone-parent families.

^m^ 64.1% of all rehospitalizations are originated in the ED.

^n^ Area-based SES quintile: an index of seven components based on American Community Survey (education index, percent persons above 200% poverty line, percent persons with a blue collar job, percent persons employed, median rental, median value of owner-occupied housing unit, and median household income).

^o^ Patients who visited an ED with chest pain or cardiac conditions were released from the ED, subsequently returned to a hospital within 0 to 7 days, and were admitted with a principal diagnosis of AMI.

^p^ Based on the classification of the American Association for the Surgery of Trauma, which encompass 621 unique ICD-9-CM.

^q^ A reading recognition test comprised 66 health-related words arranged in ascending order of difficulty.

^r^ A set of seven self-reported financial concerns items: “insurance won't cover care,” “the respondent will have to pay more than expected,” “he/she will have to pay more than he/she can afford,” “medications will cost too much,” “not being sure about being dropped from the public healthcare program,” “not knowing what the health plan covers,” and “not knowing where to go with questions about coverage.”

^s^ Seven self-reported nonfinancial barriers, including transportation difficulties, problems making appointments, not knowing where go for care, work/family responsibilities, office/clinics not being open at suitable times, obtaining childcare, and not being able to utilize one's preferred provider.

AMI, acute myocardial infarction; CCG, Clinical Commissioning Groups; CI, confidence interval; CT, census tract; CVD, cardiovascular disease; DA, dissemination area; DNR, do not resuscitate; EGS, emergency general surgery; FTR, failure to respond; HMO, Health maintenance organization; IMD, index of multiple deprivation; INSPQ, Institut national de la santé publique du Québec; IRR, incidence rate ratio; LSOA, Lower Super Output area; ON-MARG, Ontario Marginalization Index; PCI, Percutaneous coronary intervention; PCS, primary care sensitive; PPM, permanent pacemaker; RCOP, Reshaping Care for Older People; REALM, Rapid Estimate of Adult Literacy in Medicine; RR, rate ratio; SES, socioeconomic status; SIMD, Scottish Indicator of Multiple deprivation; STEMI, ST-segment elevation myocardial infarction.

#### Insurance status

Insurance coverage was used in 16 articles. Some of them compared outcomes between uninsured and insured individuals,^24,30^ between publicly and privately insured individuals,^33,38–40,46,49^ or between uninsured, publicly, and privately insured individuals.^23,25,35,41–43,47,48^

#### Social deprivation (indices of area deprivation)

This SEDH was composed of different indices, including the “Index of Multiple Deprivation,”^††^^,10,44,45^ “Carstairs Index,”^‡‡^^,31,36^ “Index of Marginalization area,”^§§^^,27^ “INSPQ deprivation Index,”^***^^,28,34^ “area-based socioeconomic status quintile index,”^†††^^,48^ and “CT/10.”^‡‡‡^^,26^

#### Income

To measure income differences, four studies used median income household (divided into quartiles or thirds),^41,43,46,47^ and one used presence versus absence of a reportable income.^50^

#### Education level

Depending on the studies, the education level was divided into three or four categories ranging from never attended school to graduate degree.^37,49^

#### Social class

This SEDH is defined hierarchically into six classes.^§§§^ It was used in one study.^31^

#### Health literacy

In one study, health literacy was the SEDH used in the health equity-focused analysis, based on scores obtained through the Rapid Estimate of Adult Literacy in Medicine test.^****^^,32^

#### Financial and nonfinancial barriers

In one article, these two types of barriers were used based on subjects' responses to 14 questions relating to financial concerns^††††^ and nonfinancial barriers.^‡‡‡‡^^29^

### Addressing health care equity through the association of emergency care indicators and SEDH

Across the studies, all identified SEDH were found to be associated with statistically significant differences in emergency care indicators. Descriptive examples of associations between equity indicators and some of the two main SEDH identified in this review are presented below (see Table 2 for details).

#### Health insurance

In a large retrospective study, including over 2.2 million patients, Lines et al. demonstrated that patients with public insurance are 2.5 times more likely to have preventable ED visits (Group 1) than private patients (rate ratio 2.53, 95% confidence interval [CI] 2.49–2.56).^33^ Similarly, in another large retrospective cohort of 1.3 million patients, Metcalfe et al. highlighted a statistically significant association between in-hospital mortality (Group 3) and insurance status among patients presenting to hospital with acute surgical conditions, requiring emergency surgery, whereby uninsured patients were at significantly higher risk of death than privately insured patients (odds ratio 1.28, 95% CI 1.16–1.41).^42^

However, some studies do not show significant differences in access or quality of care based on insurance coverage.^38,41^ Furthermore, among the studies comparing patients with and without insurance coverage, two have shown an increase in the use of ED (Group 1) after the introduction of public insurance coverage for previously uninsured patients. For example, DeLeire et al. found an increase in total ED visits (Group 1) of 46% (*p*-value, *p*<0.01) and ACSC ED visits (Group 1) of 38.7% (*p*-value, *p*<0.01) after the introduction of a public insurance (Medicaid) among low-income childless adults.^24^

Authors postulate that this may be not only due to insurance coverage increasing one's access to outpatient care but also to ED-based care. Similarly, Kerr et al., who compared ED visit rate (Group 1) among a cohort of HIV-positive patients with varying health insurance coverage (*n*=4947), showed that uninsured patients used the ED significantly less than privately insured patients (incidence rate ratio [IRR] 0.65, 95% CI 0.61–0.70), but that patients with Medicaid (public insurance program in the United States) used the ED more frequently (IRR 1.26, 95% CI 1.18–1.36).^25^

#### Social deprivation

Although social deprivation is measured by many different area-level indices among studies, it appears to be significantly associated with the three categories of indicators of emergency care identified in this review.

For example, Vanasse et al. show a relative risk of ED visits (Group 1) of *3.82* among women with mood disorders in Québec of the most deprived quintile in comparison with women of the least deprived quintile (based on an index combining social and material deprivation).^28^ Then, Lazzarino et al., who used the Carstairs Index, highlighted a significant difference in the likelihood of having a brain scan on the day of admission (Group 2) for patients presenting to the ED with an acute stroke between the least and the most deprived quartiles (odds ratio 0.94, 95% CI 0.89–0.99).^36^

Similarly, Thorne et al. demonstrate a significant association between 30-day mortality (Group 4) after ED admissions for hip fracture and social deprivation quintile with patients in the most deprived quintile at higher risk than those in the least deprived quintile, based on the Index of Multiple Deprivation (odds ratio 1.19, 95% CI 1.15–1.23).^45^

## Discussion

**FIG. 2.**
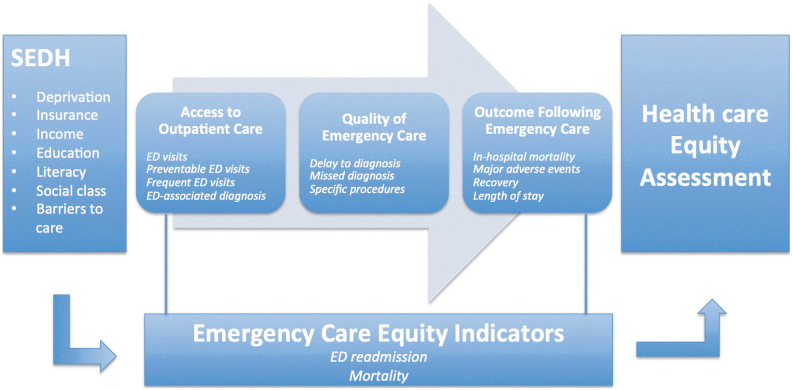
Conceptual model of Assessment of Health care Equity Representation of a conceptual synthesis of the assessment of health care equity in an emergency setting, through the combination of SEDH with emergency care equity indicators. SEDH, socioeconomic determinants of health.

Findings of this systematic review, which identified 14 health equity indicators and 7 SEDH, suggest that administrative data allow for a broad analysis of health care equity in emergency care settings. Using these health equity indicators, each of which measure different aspects of the patient pathway through emergency care, in combination with various SEDH described, presents a promising way forward in conducting health equity analyses of health care systems. Based on these findings, we have created a conceptual framework for assessing health care equity, combining SEDH through different categories of emergency care indicators, depicted in Figure 2.

The most frequently used indicator is ED visits/emergency admissions, but due to its lack of specificity, it must be interpreted with caution as there are notably many factors that could explain differences in ED visits or emergency admissions beyond health care equity, particularly differences in general health status and prevalence of diseases.^51^ ACSC ED visits/ACSC emergency admissions are arguably more specific as it focuses on ED visits/admissions that are potentially preventable with good access to primary care.^15,52^

The indicators comprising Group 2 (indicators of quality of emergency care) directly analyze emergency care and are therefore more specific in their measurement of health care equity in emergency care settings compared to indicators in Group 1. We found that they are used considerably less. This may reflect difficulty in obtaining relevant data to measure these indicators through administrative datasets. However, they might be useful indicators to use in future studies analyzing health care equity.

Among outcome indicators (Group 3), in-hospital mortality seems to be the most reproducible and available administrative data-derived indicator.

Finally, 30-/90-/326-day mortality and ED readmission, which are more global equity indicators (Group 4), assess not only the lack of access to outpatient care following an ED visit but also potential issues during the emergency care that lead to inequities in health outcome.

Due to the inherent difficulties of measuring a complex concept like health care equity and the large number of potential confounding factors, using a combination of indicators instead of one sole indicator to measure health care equity in any given health care context is more likely to result in a well-rounded assessment. As such, we suggest combining indicators across the different groups when assessing health care equity. The choice of specific indicators will depend on the context of the study, the study objectives and availability of administrative data (and relevant variables) in the health care setting of interest.

### Health equity implications

An important implication of our research is the identification of four groups of indicators that can be used to analyze equity in emergency care of high-income countries. As most of the indicators identified in this review are not specific to emergency care settings, it seems possible to study health care equity in other areas of the health care system of high-income countries with similar administrative data-derived indicators, as for example, hospitalization,^53,54^ ACSCs during the total hospital admission,^55^ and wait times.^52^ Such information could be useful for policy makers or health equity researchers to fill the gap in data about health care equity within different health care settings, particularly in high-income countries, using available administrative data.

Our findings suggest that SEDH such as insurance status or social deprivation (measured by area-based indices or median income) have a considerable impact on health care equity. The next step would also be to better characterize root causes for differences in emergency care utilization that lie outside the health care system.

For example, in a recent study, McCormick et al. demonstrate that emergency admissions are primarily due to a higher prevalence of illness in disadvantaged areas,^51^ while Pollack et al. who analyzed the relationship between neighborhood poverty and ED use in a 21-year randomized social experiment did not find a consistently significant connection between neighborhood poverty and ED use.^56^ More studies like these are needed to improve our understanding of the complex interconnectedness between SEDH, health care use, and health care equity.

### Limitations

Our review has some limitations that require consideration. First, the content and quality of administrative datasets are highly variable within countries (sometimes even within regions) and between countries. As such, many of the indicators identified in our review might not be available in many health care settings, reducing their generalizability and widespread applicability. However, important equity indicators such as preventable ED visits are frequently used and easily replicable between countries.

Second, administrative data are not designed for the purpose of equity monitoring, which implies a lack of robust quality control of the collected data, a time lag in data availability, differences in concepts and definitions used between datasets limiting comparability, and the possibility of missing records. To address this, further studies of health equity indicators and SEDH using different types of datasets would be helpful for the researchers.

Third, to define the criteria relevant to this review, it was necessary to make many normative choices before data analysis. Our focus has been indeed solely on SEDH and their associated inequities. It would also be important to analyze equity, in complementary studies, through determinants of health such as race/ethnicity, gender, or place of residence, to have a comprehensive picture of health care equity. As such, these results must be interpreted in the context of the concept of health care equity and the definitions we used. Finally, as more than half the studies were conducted in the United States, the extrapolation of the results should be carefully interpreted.

## Conclusion

Measuring health care equity should be an integral component of all comprehensive assessments of a health care system's performance. However, to measure health care equity, indicators for making such measurements need to be identified, as was the goal of this review. Such indicators can be used by researchers and policy makers interested in measuring health care equity through thoughtful selection of the most relevant indicators defined by the local context and stated objectives. Using a combination of indicators is likely to lead to a more comprehensive, well-rounded analysis of health care equity than using any one indicator in isolation.

Although studies analyzed focused on emergency care settings, it seems possible to extrapolate these indicators to measure equity in other areas of the health care system. Meta-analyses focusing on specific SEDH such as health insurance coverage, income, or indices of social deprivation in combination with studies analyzing factors that could influence the use of emergency care related to social inequalities would help to further characterize root causes of ongoing health care inequity in health care systems.

## Institutional Review Board Statement

Due to the design of the study (systematic review of the literature), no data involving participants were collected. IRB is therefore not applicable.

## Supplementary Material

Supplemental data

Supplemental data

Supplemental data

Supplemental data

## Abbreviations Used

ACSCs: ambulatory care sensitive conditions
AMI: acute myocardial infarction
CCG: Clinical Commissioning Groups
CI: confidence interval
CT: census tract
DA: dissemination area
DNR: do not resuscitate
ED: emergency department
EGS: emergency general surgery
FTR: failure to respond
IMD: index of multiple deprivation
INSPQ: Institut national de la santé publique du Québec
IRR: incidence rate ratio
LOS: length of stay
LSOA: Lower Super Output area
MAEs: major adverse events.
ON-MARG: Ontario Marginalization Index
PCI: percutaneous coronary intervention
PCS: primary care sensitive
PPM: permanent pacemaker
RCOP: Reshaping Care for Older People
REALM: Rapid Estimate of Adult Literacy in Medicine
RR: rate ratio
SEDH: socioeconomic determinants of health
SES: socioeconomic status
SIMD: Scottish Indicator of Multiple deprivation
STEMI: ST-segment elevation myocardial infarction

## Notes

^*^
Free online systematic review management system.

^†^
Including information about the design of the study, population, type of data, indicators of health care equity, SDEH addressed, main findings, and key conclusions.

^‡^
For the purpose of this article, the term “emergency admissions” is referring to a hospital admission following ED-based care or to a hospital admission for an emergency condition.

^§^
ACSCs are conditions for which it is believed that timely and appropriate outpatient care could prevent disease complications, or worsening of disease conditions, thereby preventing ED visits and hospital admissions.

^**^
Including cerebrovascular accident, pneumonia, pulmonary embolus, acute respiratory distress syndrome, renal failure, urinary tract infection, myocardial infarction, sepsis, septic shock, and cardiac arrest.

^††^
A composite score originates from the following domain indices: income, employment, health, education, access to services, community safety, and physical environment.

^‡‡^
An index of deprivation used in spatial epidemiology, based on four variables (male unemployment, lack of car ownership, overcrowding, and low social class).

^§§^
A validated census- and geography-based index that measures marginalization at the level of the census dissemination area (DA), including economic, ethnoracial, age-based, and social marginalization.

^***^
Institut national de la santé publique du Québec (INSPQ) deprivation index: an index based on six socioeconomic indicators calculated at the DA level. This index has two components, material and social. The material component is based on the proportion of people without a high school diploma, the employment-to-population ratio, and the average income. The social component is based on the proportion of people living alone, the proportion of separated, divorced, or widowed people, and the proportion of lone-parent families

^†††^
Area-based SES quintile: an index of seven components based on American Community Survey (Education index, percent persons above 200% poverty line, percent persons with a blue collar job, percent persons employed, median rental, median value of owner-occupied housing unit, and median household income).

^‡‡‡^
CT/10: a coefficient that refers to the effect of a 10% increase in the percentage of the population in the Census tract (CT), who have household incomes below 200% of the federal poverty threshold. (The poverty coefficient indicates the effect of a 10% increase in the fraction of the population living in poverty).

^§§§^
Professional, managerial, skilled nonmanual, skilled manual, semiskilled manual, and nonskilled manual.

^****^
A reading recognition test comprised 66 health-related words arranged in ascending order of difficulty.

^††††^
A set of seven self-reported financial concerns items: “insurance won't cover care,” “the respondent will have to pay more than expected,” “he/she will have to pay more than he/she can afford,” “medications will cost too much,” “not being sure about being dropped from the public healthcare program,” “not knowing what the health plan covers,” “and not knowing where to go with questions about coverage.”

^‡‡‡‡^
Seven self-reported nonfinancial barriers, including transportation difficulties, problems making appointments, not knowing where go for care, work/family responsibilities, office/clinics not being open at suitable times, obtaining childcare, and not being able to utilize one's preferred provider.
