# Supplementary material for: Measuring Health Equity in Emergency Care Using Routinely Collected Data: A Systematic Review
Source: Health Equity. 2021 Dec 1;5(1):801–17. doi: 10.1089/heq.2021.0035 (PMC8742300; doi:10.1089/heq.2021.0035)
Supplement: Supplemental data [file Supp_FileS1.pdf]

National Institute for Health Research

UNIVERSITY of York
Centre for Reviews and Dissemination

Systematic review

1. * Review title.

Give the working title of the review, for example the one used for obtaining funding. Ideally the title should state succinctly the interventions or exposures being reviewed and the associated health or social problems. Where appropriate, the title should use the PI(E)COS structure to contain information on the Participants, Intervention (or Exposure) and Comparison groups, the Outcomes to be measured and Study designs to be included.

Measuring inequity in using routinely collected hospital data: a systematic review and meta-analysis

2. Original language title.

For reviews in languages other than English, this field should be used to enter the title in the language of the review. This will be displayed together with the English language title.

3. * Anticipated or actual start date.

Give the date when the systematic review commenced, or is expected to commence.

01/11/2018

4. * Anticipated completion date.

Give the date by which the review is expected to be completed.

30/05/2019

5. * Stage of review at time of this submission.

Indicate the stage of progress of the review by ticking the relevant Started and Completed boxes. Additional information may be added in the free text box provided.
Please note: Reviews that have progressed beyond the point of completing data extraction at the time of initial registration are not eligible for inclusion in PROSPERO. Should evidence of incorrect status and/or completion date being supplied at the time of submission come to light, the content of the PROSPERO record will be removed leaving only the title and named contact details and a statement that inaccuracies in the stage of the review date had been identified.
This field should be updated when any amendments are made to a published record and on completion and publication of the review. If this field was pre-populated from the initial screening questions then you are not able to edit it until the record is published.

The review has not yet started: No

Review stage	Started	Completed
Preliminary searches	Yes	Yes
Piloting of the study selection process	Yes	No
Formal screening of search results against eligibility criteria	Yes	No
Data extraction	Yes	No
Risk of bias (quality) assessment	No	No
Data analysis	Yes	No

Provide any other relevant information about the stage of the review here (e.g. Funded proposal, protocol not yet finalised).

6. * Named contact.

The named contact acts as the guarantor for the accuracy of the information presented in the register record.

Kevin Morisod

Email salutation (e.g. "Dr Smith" or "Joanne") for correspondence:

Mr Morisod

7. * Named contact email.

Give the electronic mail address of the named contact.

kevin.morisod@unil.ch

8. Named contact address

Give the full postal address for the named contact.

9. Named contact phone number.

Give the telephone number for the named contact, including international dialling code.

0796237046

10. * Organisational affiliation of the review.

Full title of the organisational affiliations for this review and website address if available. This field may be completed as 'None' if the review is not affiliated to any organisation.

University of Lausanne and Unisanté

Organisation web address:

11. * Review team members and their organisational affiliations.

Give the personal details and the organisational affiliations of each member of the review team. Affiliation

refers to groups or organisations to which review team members belong. **NOTE: email and country are now mandatory fields for each person.**

Mr Kevin Morisod. Unisanté and University of Lausanne
Dr Xhyljeta Luta. Unisanté
Professor Patrick Bodenmann. Unisanté and University of Lausanne
Professor Joachim Marti. Unisanté and University of Lausanne

12. * Funding sources/sponsors.

Give details of the individuals, organizations, groups or other legal entities who take responsibility for initiating, managing, sponsoring and/or financing the review. Include any unique identification numbers assigned to the review by the individuals or bodies listed.

Unisanté

Grant number(s)

13. * Conflicts of interest.

List any conditions that could lead to actual or perceived undue influence on judgements concerning the main topic investigated in the review.

None

14. Collaborators.

Give the name and affiliation of any individuals or organisations who are working on the review but who are not listed as review team members. **NOTE: email and country are now mandatory fields for each person.**

Mr Thomas Bräuchli. Unisanté

15. * Review question.

State the question(s) to be addressed by the review, clearly and precisely. Review questions may be specific or broad. It may be appropriate to break very broad questions down into a series of related more specific questions. Questions may be framed or refined using PI(E)COS where relevant.

How to measure inequity in using routinely collected hospital data in the perspective of emergency?

16. * Searches.

State the sources that will be searched. Give the search dates, and any restrictions (e.g. language or publication period). Do NOT enter the full search strategy (it may be provided as a link or attachment.)

A research librarian will perform systematic searches using Ovid MEDLINE, EMBASE, PubMed and Web of Science. Searches will be limited to English-German-French-Italian- language journal articles published between 01 January 2010 and January 2019 using keywords in the field of equity, socioeconomic factors and emergency. Initial searches were conducted in January to assess the scope of the literature.

Publications will be selected in two stages. First, two reviewers are going to screen independently and in duplicate all titles and abstracts identified from searches using the inclusion and exclusion criteria. A third and fourth reviewers will provide arbitration in the event of disagreement. Second, the same reviewers will assess independently and in duplicate full text of potentially relevant articles to determine the eligibility. Any disagreement will be resolved by discussion and by consulting the third and fourth reviewer. Reasons for exclusion of articles will be documented.

Reference lists of included reviews will be screened for additional relevant studies. In addition, we will also

perform Google and Google Scholar searches using key search terms.

17. URL to search strategy.

Give a link to a published pdf/word document detailing either the search strategy or an example of a search strategy for a specific database if available (including the keywords that will be used in the search strategies), or upload your search strategy.Do NOT provide links to your search results.

Alternatively, upload your search strategy to CRD in pdf format. Please note that by doing so you are consenting to the file being made publicly accessible.

Do not make this file publicly available until the review is complete

18. * Condition or domain being studied.

Give a short description of the disease, condition or healthcare domain being studied. This could include health and wellbeing outcomes.

Equity in health (The absence of systematic or potentially remediable differences in health status, access to healthcare and health-enhancing environments, and treatment in one or more aspects of health across populations or population groups defined socially, economically, demographically or geographically within and across countries)

19. * Participants/population.

Give summary criteria for the participants or populations being studied by the review. The preferred format includes details of both inclusion and exclusion criteria.

We include studies considering adults (aged 18 and over). If a study includes both children and adults, we will limit extraction to adults. We will consider studies about every disease including for example cancer, chronic diseases (diabetes, hypertension) or mental health.

20. * Intervention(s), exposure(s).

Give full and clear descriptions or definitions of the nature of the interventions or the exposures to be reviewed.

We will focus on outcomes that are potential markers of inequity in health care, due to, for instance problems of access to primary care, poor care continuity, etc.

Examples of potential outcomes are:

Hospitalization rate, use of emergency care, emergency hospital admission, ED readmissions within 30 days, ambulatory care sensitive conditions emergency admission, specific procedures (e.g. reperfusion therapy catheterization, brain-imaging scan rate, emergency and elective caesarean section) or mortality rate.

21. * Comparator(s)/control.

Where relevant, give details of the alternatives against which the main subject/topic of the review will be compared (e.g. another intervention or a non-exposed control group). The preferred format includes details of both inclusion and exclusion criteria.

Not applicable

22. * Types of study to be included.

Give details of the types of study (study designs) eligible for inclusion in the review. If there are no restrictions on the types of study design eligible for inclusion, or certain study types are excluded, this should be stated. The preferred format includes details of both inclusion and exclusion criteria.

We will include published retrospective, observational or descriptive studies, review validation studies reports and grey literature that will report on indicators of inequities measured at the hospital level. We will include studies focusing on emergency department or emergency/unplanned care using administrative/claims data.

We plan to include data sources on indicators that are potential markers of inequity, and that are analysed as such, with a focus on emergency/unplanned hospital care in high-income countries, in order to have reproducible data for the Swiss health system.

If some relevant clinical studies are sufficiently reproducible, we will conduct a meta-analysis of them.

23. Context.

Give summary details of the setting and other relevant characteristics which help define the inclusion or exclusion criteria.

Studies in hospital services and departments. Research in high-income countries only will be included.

24. * Main outcome(s).

Give the pre-specified main (most important) outcomes of the review, including details of how the outcome is defined and measured and when these measurement are made, if these are part of the review inclusion criteria.

We will focus on outcomes that are potential markers of inequity in health care, due to, for instance problems of access to primary care, poor care continuity, etc.

Examples of potential outcomes are:

Hospitalization rate, use of emergency care, emergency hospital admission, ED readmissions within 30 days, ambulatory care sensitive conditions emergency admission and mortality rate. All these outcomes are measured using hospital administrative data.

* Measures of effect

Please specify the effect measure(s) for you main outcome(s) e.g. relative risks, odds ratios, risk difference, and/or 'number needed to treat.

Not applicable

25. * Additional outcome(s).

List the pre-specified additional outcomes of the review, with a similar level of detail to that required for main outcomes. Where there are no additional outcomes please state 'None' or 'Not applicable' as appropriate to the review

Specific procedures (e.g. reperfusion therapy catheterization, brain-imaging scan rate, emergency and elective caesarean section). All these outcomes are measured using hospital administrative data.

* Measures of effect

Please specify the effect measure(s) for you additional outcome(s) e.g. relative risks, odds ratios, risk difference, and/or 'number needed to treat.

Not applicable

26. * Data extraction (selection and coding).

Describe how studies will be selected for inclusion. State what data will be extracted or obtained. State how this will be done and recorded.

Two authors will extract data independently and in duplicate from included studies using a predefined extraction form and any discrepancies will be resolve by consulting a third and fourth reviewer. Data on the key characteristics of the studies will be extracted. This included information about the aim of the study, design, population, setting, socioeconomic indicators, main findings and conclusions.

27. * Risk of bias (quality) assessment.

Describe the method of assessing risk of bias or quality assessment. State which characteristics of the studies will be assessed and any formal risk of bias tools that will be used.

Because most of the studies included in this review are retrospective cohorts based on administrative data, the degree of evidence is moderate. In addition, there is risk of publication bias, especially since these studies focus on the same subject.

28. * Strategy for data synthesis.

Provide details of the planned synthesis including a rationale for the methods selected. This **must not be generic text** but should be **specific to your review** and describe how the proposed analysis will be applied to your data.

We will provide a narrative synthesis of the findings from the included studies based on the different type of outcome. If the included studies are sufficiently homogenous, we will provide a quantitative synthesis, using STATA software. We will use standardized mean differences for continuous variables. To check between-study heterogeneity, we will use I^2 value and funnel plot to check publication bias. In order to complete our meta-analysis, we will seek the assistance of a statistician.

29. * Analysis of subgroups or subsets.

State any planned investigation of ‘subgroups’. Be clear and specific about which type of study or participant will be included in each group or covariate investigated. State the planned analytic approach.

This is a qualitative synthesis and while subgroup analyses may be undertaken it is not possible to specify the groups in advance

30. * Type and method of review.

Select the type of review and the review method from the lists below. Select the health area(s) of interest for your review.

Type of review

Cost effectiveness
No

Diagnostic
No

Epidemiologic
No

Individual patient data (IPD) meta-analysis
No

Intervention
No

Meta-analysis
Yes

Methodology
No

Narrative synthesis
Yes

Network meta-analysis
No

Pre-clinical
No

Prevention
No

Prognostic
No

Prospective meta-analysis (PMA)
No

Review of reviews
No

Service delivery
No

Synthesis of qualitative studies
No

Systematic review
Yes

Other
No

Health area of the review

Alcohol/substance misuse/abuse
No

Blood and immune system
No

Cancer
No

Cardiovascular
No

Care of the elderly
No

Child health
No

Complementary therapies
No

COVID-19
No

Crime and justice
No

Dental

No

Digestive system
No

Ear, nose and throat
No

Education
No

Endocrine and metabolic disorders
No

Eye disorders
No

General interest
No

Genetics
No

Health inequalities/health equity
Yes

Infections and infestations
No

International development
No

Mental health and behavioural conditions
No

Musculoskeletal
No

Neurological
No

Nursing
No

Obstetrics and gynaecology
No

Oral health
No

Palliative care
No

Perioperative care
No

Physiotherapy
No

Pregnancy and childbirth
No

Public health (including social determinants of health)
Yes

Rehabilitation
No

Respiratory disorders
No

Service delivery
No

Skin disorders
No

Social care
No

Surgery
No

Tropical Medicine
No

Urological
No

Wounds, injuries and accidents
No

Violence and abuse
No

31. Language.

Select each language individually to add it to the list below, use the bin icon to remove any added in error.
English

There is not an English language summary

32. * Country.

Select the country in which the review is being carried out from the drop down list. For multi-national collaborations select all the countries involved.

Switzerland

33. Other registration details.

Give the name of any organisation where the systematic review title or protocol is registered (such as with The Campbell Collaboration, or The Joanna Briggs Institute) together with any unique identification number assigned. (N.B. Registration details for Cochrane protocols will be automatically entered). If extracted data will be stored and made available through a repository such as the Systematic Review Data Repository (SRDR), details and a link should be included here. If none, leave blank.

34. Reference and/or URL for published protocol.

Give the citation and link for the published protocol, if there is one

Give the link to the published protocol.

Alternatively, upload your published protocol to CRD in pdf format. Please note that by doing so you are consenting to the file being made publicly accessible.

No I do not make this file publicly available until the review is complete

Please note that the information required in the PROSPERO registration form must be completed in full even if access to a protocol is given.

35. Dissemination plans.

Give brief details of plans for communicating essential messages from the review to the appropriate audiences.

Do you intend to publish the review on completion?

Yes

36. Keywords.

Give words or phrases that best describe the review. Separate keywords with a semicolon or new line. Keywords will help users find the review in the Register (the words do not appear in the public record but are included in searches). Be as specific and precise as possible. Avoid acronyms and abbreviations unless these are in wide use.

Systematic review; meta-analyses; health inequity; socio-economic determinants of health; disparities;

hospital administrative data

37. Details of any existing review of the same topic by the same authors.

Give details of earlier versions of the systematic review if an update of an existing review is being registered, including full bibliographic reference if possible.

38. * Current review status.

Review status should be updated when the review is completed and when it is published. For newregistrations the review must be Ongoing.
Please provide anticipated publication date

Review_Ongoing

39. Any additional information.

Provide any other information the review team feel is relevant to the registration of the review.

40. Details of final report/publication(s) or preprints if available.

This field should be left empty until details of the completed review are available OR you have a link to a preprint.

Give the link to the published review.
