# Supplementary material for: Measuring Health Equity in Emergency Care Using Routinely Collected Data: A Systematic Review
Source: Health Equity. 2021 Dec 1;5(1):801–17. doi: 10.1089/heq.2021.0035 (PMC8742300; doi:10.1089/heq.2021.0035)
Supplement: Supplemental data [file Supp_FileS3.docx]

**Research strategy**

1. **Articles of interest**

| PubMed | 29335161[uid] OR 27656734[uid] OR 26599023[uid] OR 29137869[uid] OR 30086754[uid] |
| --- | --- |

1. **Vocabulary**

| **Concept** | **Population** | **Access/ Healthcare equity** | **Medico-economic indicators** |
| --- | --- | --- | --- |
| **Free words**  Pubmed | Asylum seeker  Ethnic minorities  Gender minority  Immigrants  Low-income people  Minorities  Non-French speaking people  Poor people  Racial minorities  Sexual minority  Undocummented Immigrants  Unemployed people  Working poor | "access to care"[tiab] OR "care access"[tiab] OR care disparit*[tiab] OR care inequalit*[tiab] OR care inequit*[tiab] OR disparities[ti] OR disparity[ti] OR equitable[ti] OR equity[ti] OR "health equity"[tiab] OR "healthcare access"[tiab] OR healthcare disparit*[tiab] OR healthcare inequalit*[tiab] OR inequalities[ti] OR inequality[ti] OR inequities[ti] OR inequity[ti] | Medical indicators of equity  Socioeconomic indicators of equity |
| **Free words**  Embase |  | 'access to care':ti,ab,kw OR disparit*:ti OR 'equity':ti OR 'healthcare access':ti,ab,kw OR 'healthcare disparit*':ti,ab,kw OR 'health care access':ti,ab,kw OR 'health care disparit*':ti,ab,kw OR 'health equity':ti,ab,kw OR 'inequalit*'.ti. OR 'inequit*'.ti. |  |
| **Emtree** |  | 'health care system'/exp  ….'health care access'/de  'health equity'/de [2016] |  |
| **MeSH** | Refugees/ OR  Sexual and Gender Minorities/ OR  Working Poor/ OR…  Emigrants and Immigrants  ...Undocumented Immigrants  Minority Groups/  Transients and Migrants/ | ("Health Services Accessibility"[Mesh:NoExp] OR "Health Equity"[Mesh] OR "Healthcare Disparities"[Mesh])  Delivery of Health Care  [Health Services Accessibility](https://www.ncbi.nlm.nih.gov/mesh/68006297)  Health Equity  OR  …..[Health Care Disparities](https://www.ncbi.nlm.nih.gov/mesh/68054625) *UF Health care inequality* | [Socioeconomic Factors](https://www.ncbi.nlm.nih.gov/mesh/68012959) *Used for (UF) : Inequalities*  *Used for : Inequality* ….Poverty  Etc. |

| **Concept** | **Type of data  routine/administrative data** | **Setting  Hospital with a focus on the emergencies** |  |
| --- | --- | --- | --- |
| **Free words**  Pubmed | "administrative claims"[tiab] OR "administrative data"[tiab] OR "administrative healthcare data"[tiab] OR "administrative health care data"[tiab] OR "administrative health data"[tiab] OR "claims-based data"[tiab] OR "claims data"[tiab] OR "discharge data"[tiab] OR "hospital billing data"[tiab] OR "hospital data"[tiab] OR "hospitalisation data"[tiab] OR "hospitalization data"[tiab] OR insurance claim*[tiab] OR "insurance data"[tiab] OR "linked data"[tiab] OR "physician billing data"[tiab] OR "routine data"[tiab] | Hospital* |  |
| **Emtree** |  | 'hospital'/exp OR 'hospital management'/exp |  |
| **MeSH** | "Insurance Claim Review"[Mesh]  [Insurance Claim Review](https://www.ncbi.nlm.nih.gov/mesh/68007345) | **milieu hospitalier** "Hospitals"[Mesh] OR "Hospital Administration"[Mesh]  **aux urgences (hosp.)**  "Emergency Service, Hospital"[Mesh]  [Emergency Service, Hospital](https://www.ncbi.nlm.nih.gov/mesh/68004636) GT Emergency Medical Services  GT Hospital Departments GT2 Hospital Administration GT Trauma Centers | GT = generic term (term above the hierarchical level)  GT2 : we go up 2 hierarchical levels |

1. **Database research – strategies**

**Medline Ovid SP**

Ovid MEDLINE(R) and Epub Ahead of Print, In-Process & Other Non-Indexed Citations and Daily 1946 to January 16, 2019

(Health Services Accessibility/ OR Health Equity/ OR exp Healthcare Disparities/ OR ((access OR disparit* OR inequ*) ADJ3 (care OR healthcare)).ti,ab,kf. OR disparit*.ti. OR equitable.ti. OR equity.ti. OR health equity.ti,ab,kf. OR inequalit*.ti. OR inequit*.ti.) **AND** (Insurance Claim Review/ OR administrative claim*.ti,ab,kf. OR administrative data.ti,ab,kf. OR administrative healthcare data.ti,ab,kf. OR administrative health care data.ti,ab,kf. OR administrative health data.ti,ab,kf. OR claims-based data.ti,ab,kf. OR claims data.ti,ab,kf. OR discharge data.ti,ab,kf. OR hospital billing data.ti,ab,kf. OR hospital data.ti,ab,kf. OR hospitalisation data.ti,ab,kf. OR hospitalization data.ti,ab,kf. OR insurance claim*.ti,ab,kf. OR insurance data.ti,ab,kf. OR linked data.ti,ab,kf. OR physician billing data.ti,ab,kf. OR routine data.ti,ab,kf.) **AND** ((**(exp Hospitals/ OR exp Hospital Administration/ OR hospital*.ti,ab,kf.) AND** emergenc*. ti,ab,kf,hw.) OR Emergency Department. ti,ab,kf.) NOT (child not adult).mp. NOT (Comment or Editorial or Meeting Abstract).pt.

limit 1 to (yr="2010 - 2019" and (english or french or german or italian))

| **128** | **references 2010-2019** | **21.01.2019** |
| --- | --- | --- |

| **Comments :** | - ab = abstract ; ti = title - kf = keyword heading word - / = sh (Mesh Subject Headings) - hw = Subject heading word |
| --- | --- |
|  | Limited to  - English/German/French/Italian  - years 2010-2019 - We exclude children without adults  **NOT (child not adult).mp.** [Source : W. Bramer] N’enlève pas tout, mais déjà une partie des références pédiatriques.  - We exclude conference abstract, editorial and opinion papers.  **NOT (Comment or Editorial or Meeting Abstract).pt.** |
|  | ED can be the abbreviation of several things. We assume that the developed form is used at least in the title or abstract. With the proposed equation, we also find the articles on "Emergency Department", but which do not mention the term "hospital*". |

**PubMed**

("access to care"[tiab] OR "care access"[tiab] OR care disparit*[tiab] OR care inequalit*[tiab] OR care inequit*[tiab] OR disparities[ti] OR disparity[ti] OR equitable[ti] OR equity[ti] OR "health equity"[tiab] OR "healthcare access"[tiab] OR healthcare disparit*[tiab] OR healthcare inequalit*[tiab] OR inequalities[ti] OR inequality[ti] OR inequities[ti] OR inequity[ti]) **AND** (administrative claim*[tiab] OR "administrative data"[tiab] OR "administrative healthcare data"[tiab] OR "administrative health care data"[tiab] OR "administrative health data"[tiab] OR "claims-based data"[tiab] OR "claims data"[tiab] OR "discharge data"[tiab] OR "hospital billing data"[tiab] OR "hospital data"[tiab] OR "hospitalisation data"[tiab] OR "hospitalization data"[tiab] OR insurance claim*[tiab] OR "insurance data"[tiab] OR "linked data"[tiab] OR "physician billing data"[tiab] OR "routine data"[tiab]) **AND** ((**hospital*[tiab] AND** emergenc*[tiab]) OR "Emergency Department"[tiab]) NOT ("Comment"[Publication Type] OR "Editorial"[Publication Type] OR "Meeting Abstract"[Publication Type]) **NOT medline[sb]**

**Embase.com**

('health care access'/de OR 'health equity'/de OR 'health care disparity'/de OR ((access OR disparit* OR inequ*) NEAR/3 (care OR healthcare)):ti,ab,kw OR disparit*:ti OR equitable:ti OR equity:ti OR 'health equity':ti,ab,kw OR inequalit*:ti OR inequit*:ti) **AND** ('administrative claim*':ti,ab,kw OR 'administrative data':ti,ab,kw OR 'administrative healthcare data':ti,ab,kw OR 'administrative health care data':ti,ab,kw OR 'administrative health data':ti,ab,kw OR 'claims-based data':ti,ab,kw OR 'claims data':ti,ab,kw OR 'discharge data':ti,ab,kw OR 'hospital billing data':ti,ab,kw OR 'hospital data':ti,ab,kw OR 'hospitalisation data':ti,ab,kw OR 'hospitalization data':ti,ab,kw OR 'insurance claim*':ti,ab,kw OR 'insurance data':ti,ab,kw OR 'linked data':ti,ab,kw OR 'physician billing data':ti,ab,kw OR 'routine data':ti,ab,kw) **AND** ((**('hospital'/exp OR 'hospital management'/exp OR 'hospital*':ti,ab,kw) AND** emergenc*:ti,ab,kw,de) OR 'Emergency Department':ti,ab,kw) NOT (child NOT adult) AND ([english]/lim OR [french]/lim OR [german]/lim OR [italian]/lim) NOT ([conference abstract]/lim OR [editorial]/lim) AND [2000-2019]/py

| **127** | **references 2010-2019** | **17.01.2019** |
| --- | --- | --- |
| **Comments** | Limited to  - English/German/French/Italian  - years 2010-2019  **AND [2010-2019]/py**  - we exclude children without adults  **NOT (child NOT adult)** [Source :Wichor M. Bramer, courriel du 19.2.2018  N’enlève pas tout, mais déjà une partie des références pédiatriques.  *N.B. it could be risky to add :*  *AND ([young adult]/lim OR [adult]/lim OR [middle aged]/lim OR [aged]/lim OR [very elderly]/lim)*  - we exclude conference abstract and editorial **NOT ([conference abstract]/lim OR [editorial]/lim)** | |

**Web of Science – Core collection**

Stratégie avec syntaxe adaptée à copier/coller dans base de donnée [ici](http://apps.webofknowledge.com/WOS_AdvancedSearch_input.do?SID=N2uyoG9eUGeFr8tJwBa&product=WOS&search_mode=AdvancedSearch)

(Topic Research)

(((access OR disparit* OR inequ*) N3 (care OR healthcare)) OR disparit* OR equitable OR equity OR "health equity" OR inequalit* OR inequit*) AND ("administrative claim*" OR "administrative data" OR "administrative healthcare data" OR "administrative health care data" OR "administrative health data" OR "claims-based data" OR "claims data" OR "discharge data" OR "hospital billing data" OR "hospital data" OR "hospitalisation data" OR "hospitalization data" OR "insurance claim*" OR "insurance data" OR "linked data" OR "physician billing data" OR "routine data") AND ((hospital* AND emergenc*) OR "Emergency Department") NOT (child NOT adult)

Timespan: 2000-2019. Indexes: SCI-EXPANDED, SSCI, A&HCI, CPCI-S, CPCI-SSH, BKCI-S, BKCI-SSH, ESCI, CCR-EXPANDED, IC.

| **85** | **references 2010-2019** | **17.01.2019** |
| --- | --- | --- |
| **Comments** | The research is done by subject (TOPIC). Therefore, terms such as "equity" or "inequalit*", searched only in the title in Medline or Embase, are searched more broadly in Web of Science. This probably generates additional references that are less interesting, but it is worth checking. | |
